# Aspiration-driven co-evolution of cooperation with individual behavioral diversity

**DOI:** 10.1371/journal.pone.0291134

**Published:** 2023-09-15

**Authors:** Yongqiong Yuan, Jian Wang, Zhigang Wang, Haochun Yang, Tao Xu, Huang Huang

**Affiliations:** 1 Key Laboratory of Data Link, China Electronics Technology Group Corporation, Xi’an, China; 2 AVIC Chengdu Aircraft Design & Research Institute, Chengdu, China; 3 School of Computer Science and Engineering, Northwestern Polytechnical University, Xi’an, Shaanxi, China; Teesside University, UNITED KINGDOM

## Abstract

In evolutionary game, aspiration-driven updates and imitation updates are the two dominant game models, and individual behavior patterns are mainly categorized into two types: node player and link player. In more recent studies, the mixture strategy of different types of players has been proven to improve cooperation substantially. Motivated by such a co-evolution mechanism, we combine aspiration dynamics with individual behavioral diversity, where self-assessed aspirations are used to update imitation strategies. In this study, the node players and the link players are capable to transform into each other autonomously, which introduces new features to cooperation in a diverse population as well. In addition, by driving all the players to form specific behavior patterns, the proposed mechanism achieves a survival environment optimization of the cooperators. As expected, the interaction between node players and link players allows the cooperator to avoid the invasion of the defector. Based on the experimental evaluation, the proposed work has demonstrated that the co-evolution mechanism has facilitated the emergence of cooperation by featuring mutual transformation between different players. We hope to inspire a new way of thinking for a promising solution to social dilemmas.

## 1 Introduction

Cooperative behavior commonly exists in nature and human society [1]. From the perspective of the whole environment, spontaneous cooperative behavior is conducive to the sustainable development of the group and the maximization of the overall interests of the group [2]. But for individuals, defectors usually gain more than cooperators due to competitive relationships and limited resources, which means that selfish defection often results in a greater payoff. In the environment of fittest survival, the defection strategy seems to be an optimized solution according to the law of survival [3–6]. With the emergence of the contradiction between the individual’s defection and the group’s cooperation, as a consequence, why cooperative behavior emerges and is maintained in selfish individuals has become a widely concerned topic.

To solve the contradiction and facilitate the emergence of cooperation, different approaches based on evolutionary game theory [7, 8] have been proposed. Nowak effectively combined network reciprocity with evolutionary game theory in the ways of kin selection [9, 10], direct reciprocity [11–13], indirect reciprocity [13, 14], network reciprocity [15–17], and group selection [18]. By investigating the evolution of cooperation on complex networks, it was found that different network topologies have a significant effect on the generation of cooperative clusters [19–24]. On the other hand, the aspiration-driven dynamic [25–27] and incentive direction [28–31] are also effective methods of promoting cooperation. In imitation-driven dynamics, individuals adjust their strategies based on the gains obtained from the game compared to their aspirations. In contrast, individuals imitate the strategies of more successful peers in aspiration dynamics [32].

However, most current studies on games are based on a strict node-to-node pattern in which each node player plays with a uniform strategy towards its surrounding neighbors [33–35]. In this pattern, some limitation is still hard to be avoided, for example, a player adopts a defection strategy to defend against a defector’s invasion in the neighborhood, meanwhile, this behavior may harm nearby cooperators as a side effect. Based on the interactive heterogeneity of players [36], the “link players” are introduced into the game to adopt different strategies when playing games with different neighbors [37, 38].

Studies on link player games have shown that the node players and the link players have different properties in different network topologies [36, 39]. In structured networks, link players are able to reach higher levels of cooperation in the Prisoner’s Dilemma (PD) game [40]. Inspired by these findings, Su et al. argued that the strategies of link players evolve in the form of behavior diffusion along edges in the game [41, 42]. The discrepancy in behavioral patterns of the node player and link player leads to different evolutionary outcomes. A study by Jia et al. revealed the effect of a mixed crowd community of link players and node players on cooperation and also proved that mixed groups of link and node players enable a higher level of cooperation. When the density of link players is low, node players consciously form cooperative groups to resist defection [43].

More recently, researchers have been exploring mixed-player games involving both node players and link players, aiming to better understand co-evolution mechanisms that drive the promotion of cooperation. However, many of these studies have taken a somewhat uniform and statistical approach [43]. Drawing from insights in sociology, it’s been recognized that individual mindsets, ranging from radicalism to conservatism [44, 45], can significantly influence decision-making. In this context, conservatism refers to the inclination to maintain the existing status quo, whereas radicalism involves challenging or altering the status quo.

To address these considerations, we’ve introduced a novel perspective in our work. We’ve embraced the interconnected nature of radicalism and conservatism and combined them with aspiration-driven dynamics to create a dynamic aspiration mechanism. This innovative approach allows for a continuous exchange between node players and link players, enabling them to transition between these roles organically. Our experimental results underscore the efficacy of this mechanism, demonstrating a marked improvement in cooperation levels. Furthermore, this mechanism enhances the system’s ability to adapt to changing environments.

## 2 Materials and methods

In the proposed model, the study of repeated PD games is conducted on an *L×L* periodic lattice network [46]. The payoff of the PD is usually represented by a two-dimensional matrix as:

$$\begin{array}{cc} & \begin{array}{cc} C & D \end{array}\\ \begin{array}{c} C\\ D \end{array} & \left(\begin{array}{cc} R & S\\ T & P \end{array}\right) \end{array}$$ (1)

Suppose that two participants simultaneously decide to take cooperation C or defection D, the payoffs caused by different actions contain reward *R*, punishment *P*, temptation *T*, and a sucker’s payoff *S*. For instance, to obtain payoff *S*, the strategy from the first row *C* needs to meet the strategy from the second column *D*. The PD requires the conditions *T>R>P>S*, *2R>S+T* [47]. The values of *R*, *S*, *T*, and *P* are set to 1, 0, *b*, 0, respectively. Specifically, b is set between 1 and 2 by following a game set-up criteria called the “weak Prisoner’s Dilemma” [48].

The repeated PD game is simulated in a periodic lattice network, in which each node represents a player and the connections between them are the relationship. The player plays a PD with its surrounding neighbors by setting *L* to 100. There are two types of players in the network: node player and link player. Node players adopt a uniform strategy towards their neighboring nodes, while link players take different strategies towards different neighbors. During initialization, all players are initialized as node players, who have an equal probability to adopt a cooperation or defection strategy.

In the game, player *x* is randomly selected from all participants, then the selected player *x* randomly chooses a surrounding neighbor *y* to play to gain *P*_*xy*_. The payoff *P*_*x*_ of player *x* is defined as the sum of the gains of the game between player *x* and all surrounding neighbors, as follows:

$$P_{x}=\sum_{y\in \Omega_{x}}P_{xy}$$ (2)

where *Ω*_*x*_ is the set of four neighbors around player *x*. Players have the dynamic aspiration property, and the term ‘aspiration’ here denotes a player’s expectation of the payoff in the game. Noted that the aspiration evolves as the game evolves, the aspiration of player *x* is only related to the payoff of its own and the previous aspiration. Furthermore, aspiration can also act as an indicator of the player’s satisfaction. If player *x*’s aspiration is greater than or equal to that in the previous round, the state of *x* can be regarded as satisfied, otherwise is unsatisfied. The equation of aspiration is:

$$A_{x}(t+1)=A_{x}(t)+a*(P_{x}(t)-A_{x}(t))$$ (3)

where *P*_*x*_(*t*) denotes the payoff of player *x* in round *t*, *A*_*x*_(*t*) denotes player *x*’s aspiration in round *t*, and *a* (0<*a*≤1) indicates the player’s learning rate of aspiration (0.1 by default). Player *x*’s aspiration in round *t*+1 depends on the player *x*’s payoff and aspiration in round *t*. According to Formula (3), when the game reaches equilibrium, the type of player won’t change. So, *A*_*x*_(*t*+1) should be equal to *A*_*x*_(*t*), which means *P*_*x*_(*t*)−*A*_*x*_(*t*) should be zero. In other words, when the game reaches equilibrium, player *x*’s payoff should be equal to player *x*’s aspiration. That’s why player *x*’s aspiration converges to *p* on the condition that the payoff of player *x* is a stable value *p*. If all players adopt a cooperative strategy in the game sustainably, players will get the maximum payoff of 4. Then the value domain of aspiration is (0,4]. The aspiration of all players is initialized to 2, i.e. *A*_*x*_(0) = 2. Using aspiration, we build a set of mechanisms for node and link players to transform each other cyclically:

$$\left\{ \begin{array}{cc} N_{x}\to L_{x} & if\;N_{x}^{\prime }\;s\;A_{x}(t+1)<A_{x}(t)\\ L_{X}\to N_{x} & if\;L_{x}^{\prime }\;s\;A_{x}(t+1)\geq A_{x}(t) \end{array}\right.$$ (4)

where *N*_*x*_ denotes that player *x* is a node player and *L*_*x*_ denotes that player *x* is a link player. The mechanism of co-evolution of node players and link players is triggered after player *x* updates its strategy. In case of adversity, individuals tend to become radical and try to change the current situation. Conservative behavior is a better choice for individuals to maintain the status quo in good times. When node player *x* is dissatisfied at the time *A*_*x*_(*t*+1)<*A*_*x*_(*t*), node player *x* will be in a state of radicalism and transform into a link player. When link player *x* is satisfied at the time *A*_*x*_(*t*+1)≥*A*_*x*_(*t*), link player *x* will be in a state of conservatism and transform into a node player.

**Fig 1:**
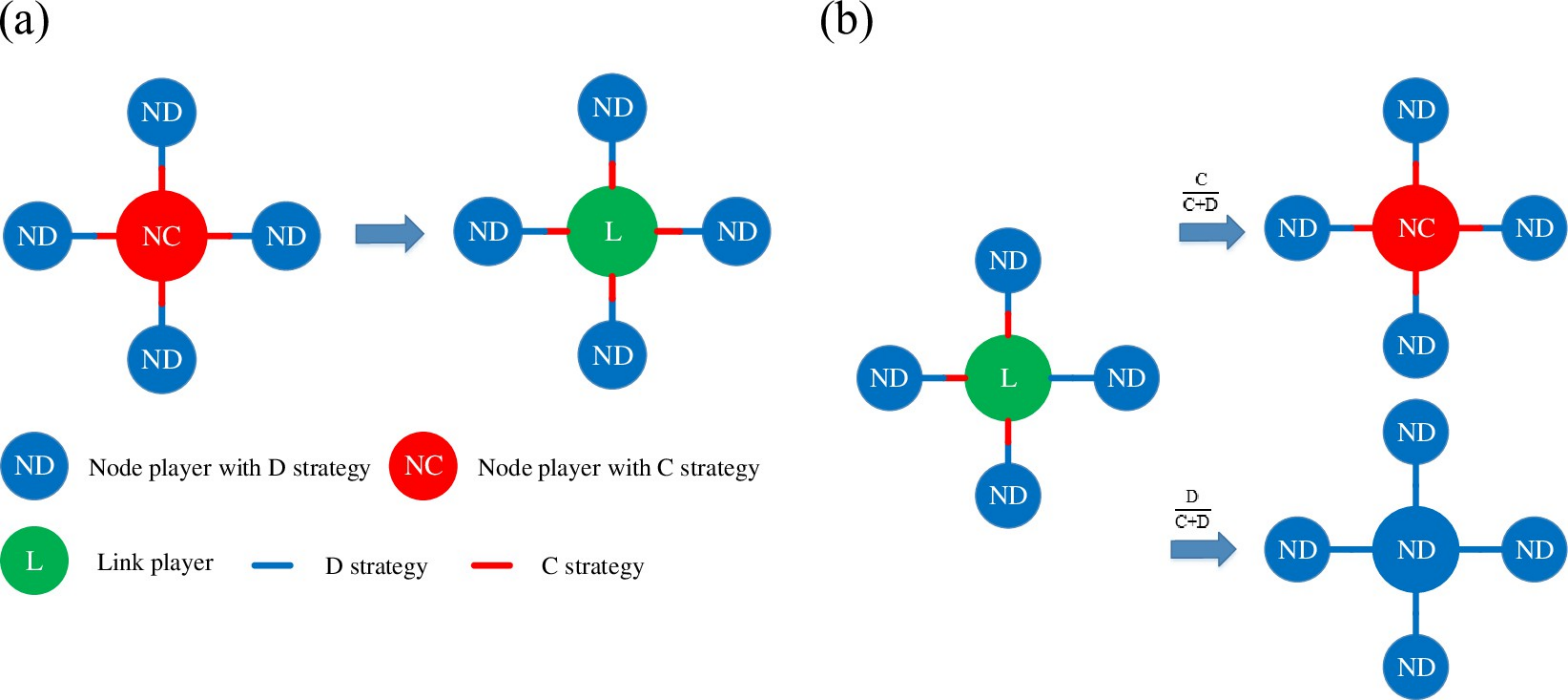
Panels (a)–(b) show the specific process of interconversion between node players and link players. (a) shows the process of converting a node player to a link player, the link player treats its surrounding neighbors uniformly with the original strategy. (b)shows the transformation of a link player to a node player. Since node player can only adopt one strategy, link player is converted into node player who adopts cooperation (defection) strategies based on the percentage of cooperation (defection) strategies.

In the process of interconversion between node players and link players, it is necessary to pay attention to some differences in the traits of node and link players. When player *x* is transformed from a node player to a link player, the strategy of handling the surrounding neighbors is uniformly changed to the original strategy. When player *x* is converted from a link player to a node player, it cannot be directly converted to a node player because of the heterogeneity of the link player’s strategy. To solve this problem, the proportion of the four edges that adopt cooperation and defection strategy is calculated respectively. This proportion is used as the probability of adopting a cooperation (defection) strategy after the link player transforms into a node player. For example, suppose that the link player *x* has four neighbors *y*_1_, *y*_2_, *y*_3_, and *y*_4_, and it adopts a cooperative strategy in the game with neighbors *y*_1_, *y*_2_, and *y*_3_, and a defection strategy in the game with the neighbor *y*_4_. After the link player *x* is transformed into a node player, the probability of cooperative strategy adaptation is $\frac{C}{C+D}=0.75$ and the probability of defection strategy adaptation is $\frac{D}{C+D}=0.25$. The detailed transformation mechanism is illustrated as Fig 1:

When the player *x* selects a neighbor for a PD game, the classic Fermi function is used to update the strategy of *x* [49].

$$W_{\left(S_{x}\leftarrow S_{y}\right)}=\frac{1}{1+\exp\left[\left(P_{x}-P_{y}\right)/K\right]}$$ (5)

The Formula (5) represents the probability that player *x* imitates player *y*′s strategy after playing with player *y*, where *P*_*x*_ and *P*_*y*_ respectively denote the payoff of players *x* and *y*, and *S*_*x*_ and *S*_*y*_ denote the strategies of players *x* and *y*. K is the random noise describing the uncertainty of the strategy update. Here, when imitation actions occur, four situations will occur. (a)If both *x* and *y* are node players, *x* will simply imitate the strategy of *y*. (b)If *x* is a node player and *y* is a link player, *x* will imitate the strategy on the link between *x* and *y*. (c)If both x and y are link players, *x* will imitate the strategy on the link between *x* and *y*. (d)If *x* is a link player and *y* is a node player, the strategy on the link between *x* and *y* will imitate the strategy of *y*. To simplify the experiment without losing generality, the *K* is set to 0.1. The Monte Carlo (MC) steps are set to 5000. To ensure correct accuracy, each set of parameters was repeated 20 times independently and the final results are averaged.

## 3 Results and analysis

To verify the effectiveness of the proposed co-evolution mechanism of node and link players, substantial simulations are conducted on the overall frequency of cooperation (*ρ*_*c*_) in the game.

**Fig 2:**
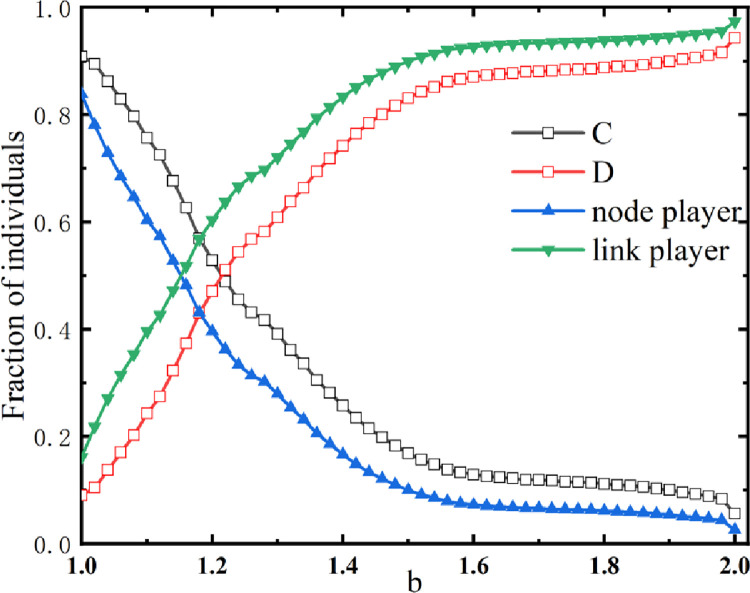
The trend of the overall frequency of cooperation and defection at different *b* values is related to the percentage of both types of players for *a* = 0.1.

Fig 2 shows the relationship between the overall frequency of cooperation (defection) and the percentage of node (link) players, both of them are influenced by the value of *b*. The overall frequency of cooperation (*ρ*_*c*_) under the co-evolution mechanism of node and link players is maintained at a high level compared to the traditional PD game. As we can see, as the value of *b* increases, the overall frequency of cooperation (*ρ*_*c*_) and the proportion of node players decrease together, but the overall frequency of defection and the proportion of link players increase together. When 1.1<*b*<1.6, *ρ*_*c*_ decreases quickly and soon remains at a certain percentage as the game becomes more difficult. If *b*>1.6, *ρ*_*c*_ slowly decreases to maintain a relatively stable state, and the co-evolution mechanism allows cooperative players to survive. The main players are composed of defectors, and cooperative players account for a relatively small percentage.

From Fig 2, it can be observed that there is a strong similarity between the trend of the percentage of node players and the trend of the cooperation frequency (*ρ*_*c*_). By the same token, the percentage of link players and the frequency of defection also reveal this phenomenon. This demonstrates that there is a relationship between the percentage of both types of players and the overall frequency of cooperation.

**Fig 3:**
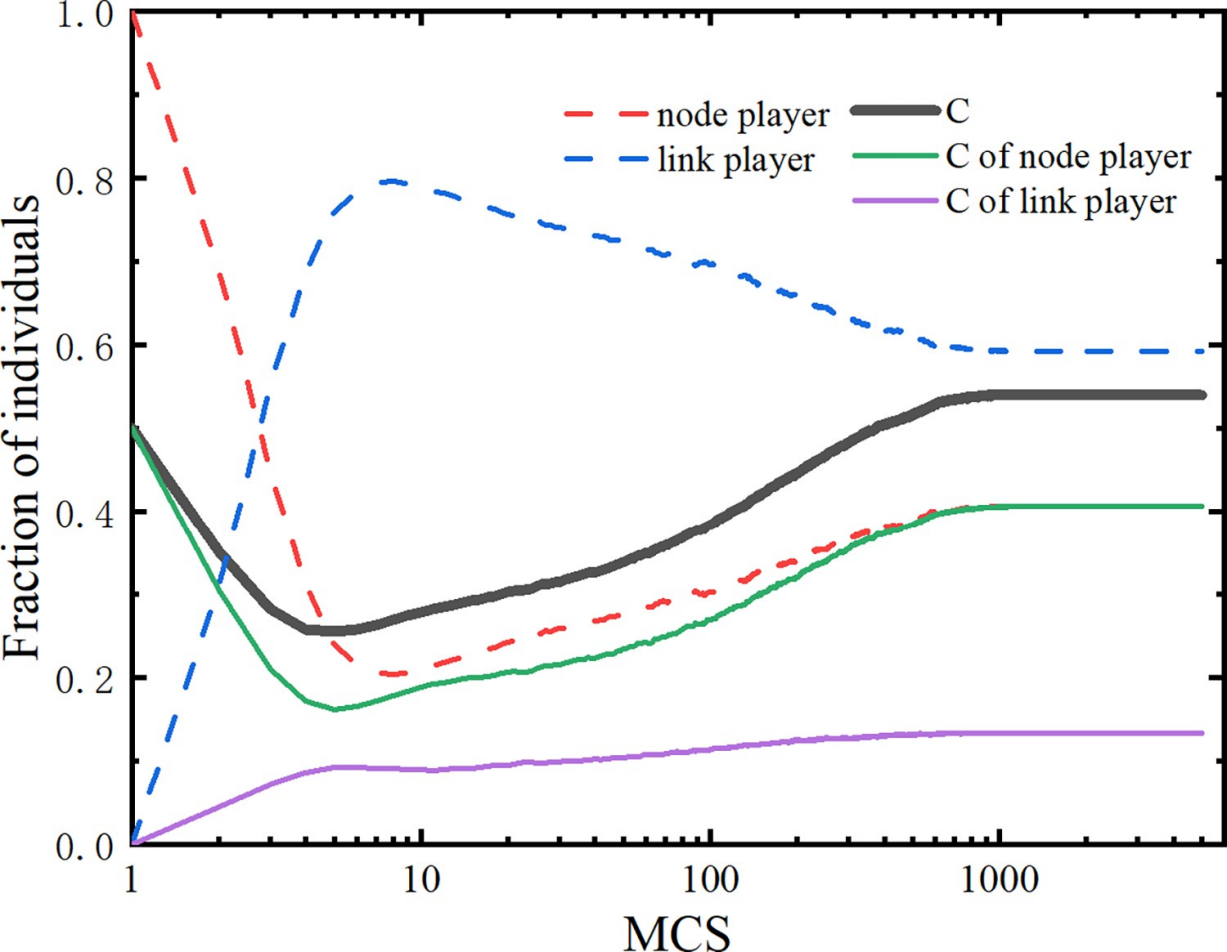
Comparison of the proportion of two types of players, the frequency of cooperation of two types of players, and the overall frequency of cooperation. All results are shown for *b* = 1.2.

To further study the relationship between the percentage of both types of players and the frequency of cooperation (*ρ*_*c*_), we also investigate the evolution of cooperative strategy among two types of players, in which the value of *b* is set to 1.2. As shown in Fig 3, the proportion of link players is rapidly increasing at the beginning because of the transformation from node players to link players. The main reason is that most node players are at a disadvantaged point in the game because of the weak survivability of node players, only a small proportion of node players survive and gradually form cooperative clusters. In the subsequent evolution, the link players around cooperative groups (composed of node players) are gradually transformed into node players, which is the result of the aspiration of link players keeping rising. The cooperation frequency of link players (*ρ*_*lc*_) is lower than that of node players (*ρ*_*nc*_), as a consequence, the link players play more of a role in promoting cooperation. Eventually, both types of players reach the equilibrium state, and the conversion between node players and link players no longer takes place.

**Fig 4:**
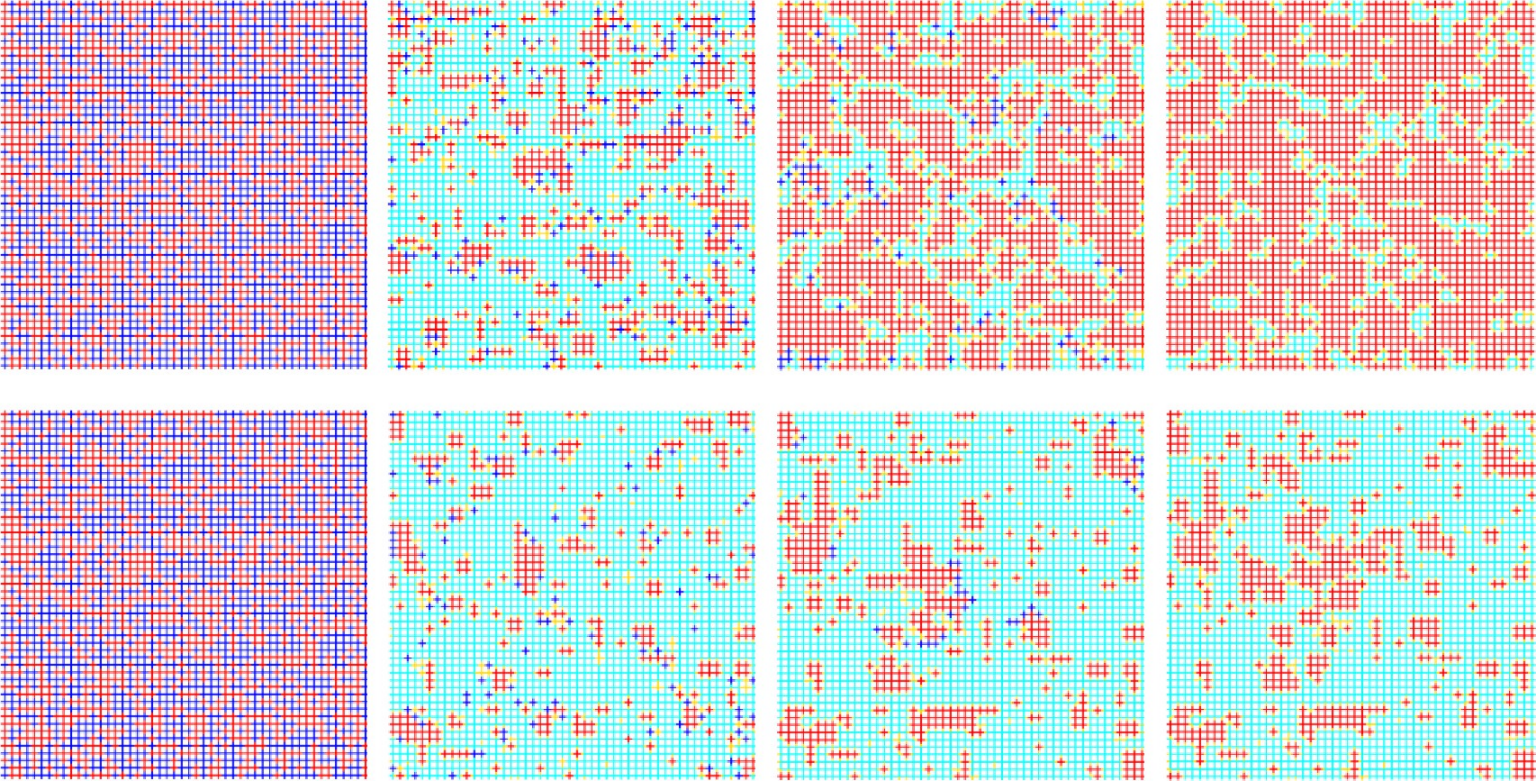
The node players with cooperation strategy survive because of the isolation zone formed by being surrounded by link players. Red, blue, gold, and cyan denote node players with cooperation strategy, node players with defection strategy, link players with cooperation strategy, and link players with defection strategy, respectively. The two rows of snapshots correspond to *b* values of 1.04 and 1.2, respectively. The images from left to right show the number of MC steps as 0, 5, 100, and 3000 respectively.

To present more effect details of the evolutionary game process, snapshots of the characteristics are taken from a microscopic perspective at different b values in Fig 4. It can be easily found that link players are not present at the beginning and then rapidly appear abundantly. At *b* = 1.04, node players disappear firstly and then reoccupy the body of the network in the subsequent evolution. But at *b* = 1.2, link players always occupy the body of the network. In the process of evolutionary game, a minority of node players with cooperation strategy gather into groups and form cooperative communities. Node players with defection strategy and link players with defection strategy gather at the edge of the group of node players with cooperation strategy. Eventually, all node players with defection strategy become extinct, the mutual transformation of node players and link players tends to balance. The link players surround the node players to form an isolation zone to ensure the survival of node players with a cooperation strategy. The game mechanism between a group of node players with cooperative strategy and players at the edge of the group is significantly different from the traditional game model, which promotes unique behavioral patterns for both types of players.

**Fig 5:**
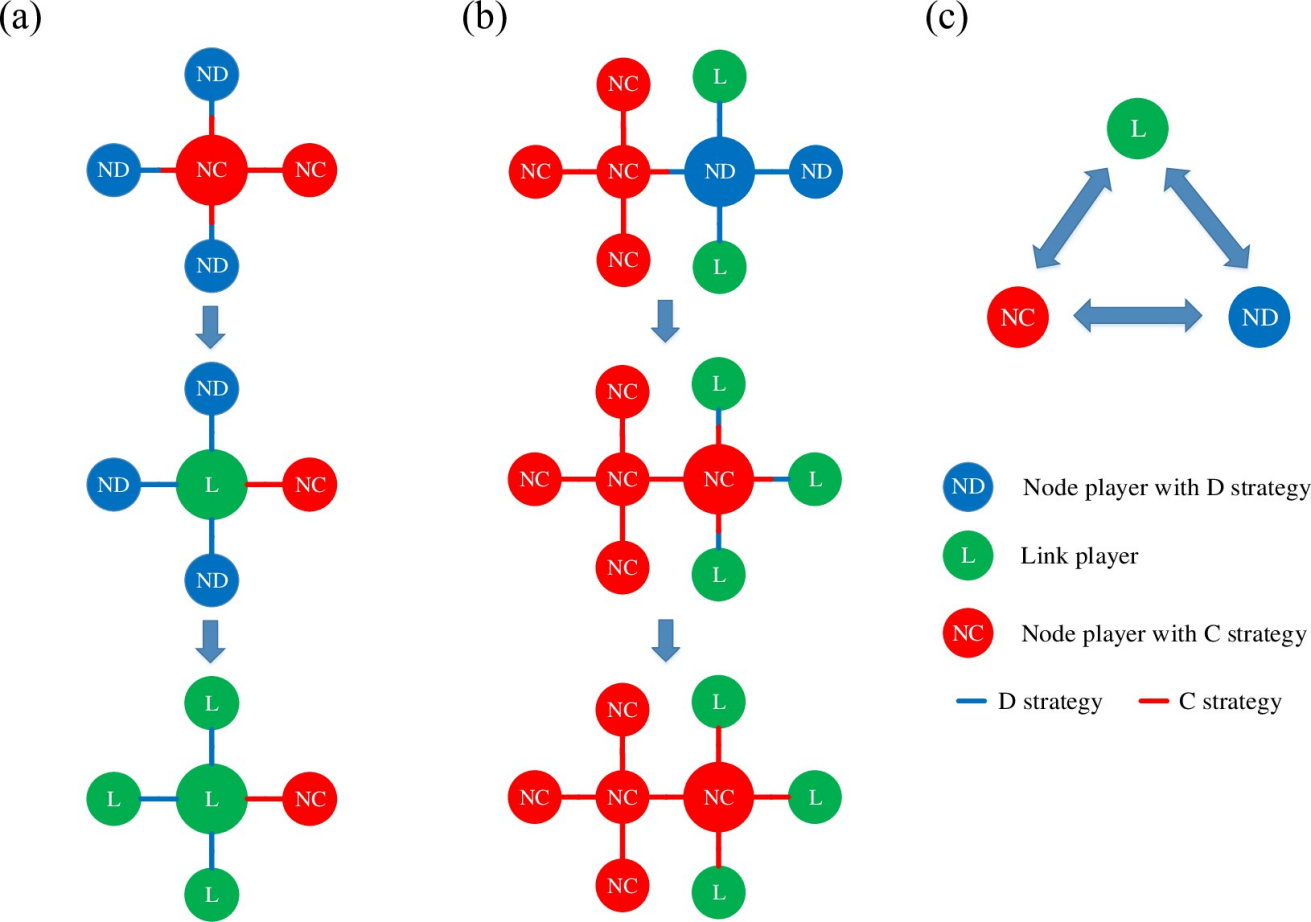
Patterns of behavior among node and link players under the co-evolution mechanism play a role in facilitating cooperation. (a) shows the behavior pattern of the node players adopting a cooperation strategy. (b)shows the behavior pattern of node players adopting the defection strategy. Node players with cooperation strategy, node players with defection strategy, and link players transform into each other as shown in (c).

The co-evolution mechanism drives node and link players to form specific behavior patterns, as shown in Fig 5. The role played by the co-evolution mechanism can be summarized in two aspects: on the one hand, to prevent the demise of the cooperator community (Fig 5(A)), and on the other hand, to promote the growth of the cooperator community (Fig 5(B)). In the process of the game, the surviving node players generally adopt a cooperation strategy. As we can see in Fig 5(A), node players are transformed into link players to obtain part of the benefits of cooperation strategy, to defend against players adopting defection strategy. The node player adopting the defection strategy loses the exploitative target and transforms into the link player because of the extinction of the node player adopting the cooperation strategy. As shown in Fig 5(B), node players adopting the defection strategy occupy the edge of the group which is composed of node players adopting the cooperation strategy, and they often do not survive for long. Some of them eventually transform into node players adopting the cooperation strategy, while others transform into link players, depending on the gain between cooperation strategy and defection strategy. There are wide variations in the frequency of cooperation at different game difficulties, but the co-evolution mechanism performs a very similar function. The co-evolution mechanism serves to protect node players with cooperation strategy, which is an important reason why node players are surrounded by link players adopting cooperation strategy when the game is in equilibrium. With the mechanism, node players with cooperation strategy and defection strategy, and link players can be transformed into each other, which makes the choice of strategy more flexible and increases the probability of survival of the cooperators.

**Fig 6:**
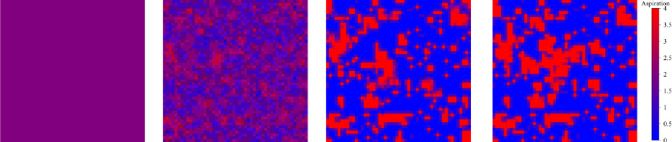
The evolution of aspiration is consistent with the evolution of cooperation, where *b* = 1.2. From left to right, the MC steps are 0, 5, 100, and 3000. The population consisting of node players with cooperation strategy overlaps with the clusters of aspiration.

The mutual transformation of different types of players is closely related to aspiration, and snapshots of the aspiration are captured in the evolution. As shown in Fig 6, it is obvious that the evolution of player aspiration is surprisingly consistent with the evolution of cooperation. The player’s aspiration reflects the player’s payoff. When evolution is in the state of equilibrium, the player’s aspiration is as same as the player’s payoff. The group clusters are formed by node players via adopting cooperation strategy, and the corresponding gain is stable at 4 in evolutionary equilibrium, which means that the aspiration of the node players in the group finally converges to 4. For the link players surrounding the node players, they have at least one adjacent edge adopting the cooperation strategy. As a result, the aspiration of link players in adjacent positions is higher than that of other link players. Based on the comparison with Fig 4(b), the aspiration and benefits are mutually verified.

**Fig 7:**
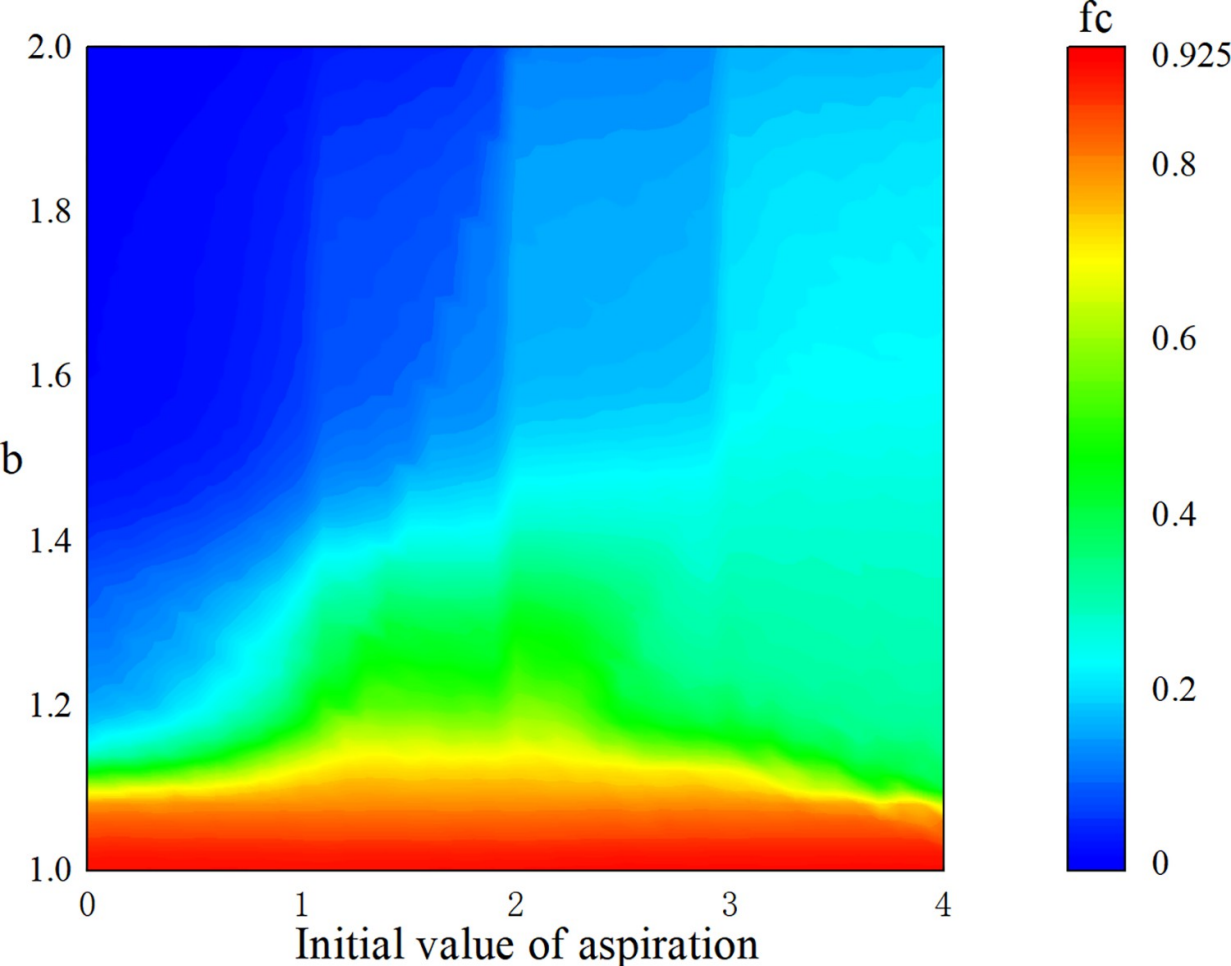
The effect of different initial values of aspiration on the frequency of cooperation. When the aspiration is initialized to 2, the frequency of cooperation reaches an extreme value.

With different initial values, the effect of aspiration on the frequency of cooperation is explored, and the results are shown in Fig 7. In the previous experiment, the aspiration is initialized to 2 (ranging in (0,4]), which may ensure the universality of the experimental results to the maximum extent. When *b*<1.1, the frequency of cooperation reaches a very high level regardless of the initial value of aspiration, and when *b*>1.1, the frequency of cooperation decreases rapidly. As the initial value of aspiration increases, the survival ability of the cooperator is improved, cooperator can maintain a certain frequency of cooperation in a hard PD environment. In addition, it can be seen that the frequency of cooperation reaches its maximum when the initial value of aspiration is set to 2.

## 4 Conclusion

In this paper, an evolutionary game system is established, where the node players and link players can be transformed into each other, to study the evolution of cooperation. Compared to the static model proposed by Jia et al., the model proposed in our study is dynamic. In our study, the node players and link players can be transformed into each other through aspirations. We introduce the aspiration inspired by the mindsets between radicalism and conservatism. Here, the aspiration acts as an indicator of the player’s satisfaction, which will evolve as the game evolves. After performing numerical simulations, we find that the level of cooperation can be effectively promoted under the proposed mechanism. The co-evolution mechanism drives node and link players to prevent the demise of the cooperator community by forming specific behavior patterns. With an isolation zone, the node players are surrounded by the link players to ensure the survival of cooperator. Moreover, there is a striking consistency in the evolution of player aspiration and cooperation. The combination of aspiration-driven dynamics and individual behavioral diversity has a particular contribution to the evolution of cooperation. The co-evolution mechanism is more in line with the characteristics of the species diversity in nature, and more closely resembles the natural evolution. We hope to attract increasing attention and interest to continue along this line of study as well.

## Supporting information

S1 Data (ZIP)

## References

[1] Smith J.M., Evolution and the Theory of Games, Cambridge university press, 1982.

[2] Margulis L., Symbiosis in cell evolution: Life and its environment on the early earth, (1981).

[3] Hofbauer J., Sigmund K., Evolutionary games and population dynamics, Cambridge university press, 1998.

[4] Manhes P., Velicer G.J., Experimental evolution of selfish policing in social bacteria, Proceedings of the National Academy of Sciences, 108 (2011) 8357–8362. doi: 10.1073/pnas.1014695108 21531905 PMC3100924

[5] Gould S J, Eldredge N. Punctuated equilibria: an alternative to phyletic gradualism. Models in paleobiology, 1972 (1972): 82–115.

[6] Wade M.J., Kalisz S., The causes of natural selection, Evolution, 44 (1990) 1947–1955. doi: 10.1111/j.1558-5646.1990.tb04301.x 28564434

[7] Nowak M.A., Five rules for the evolution of cooperation, Science, 314 (2006) 1560–1563. doi: 10.1126/science.1133755 17158317 PMC3279745

[8] Axelrod R., Hamilton W.D., The evolution of cooperation, Science, 211 (1981) 1390–1396. doi: 10.1126/science.7466396 7466396

[9] Hamilton W.D., The genetical evolution of social behaviour. II, J. Theor. Biol., 7 (1964) 17–52. doi: 10.1016/0022-5193(64)90039-6 5875340

[10] Ale S.B., Brown J.S., Sullivan A.T., Evolution of cooperation: combining kin selection and reciprocal altruism into matrix games with social dilemmas, PLoS ONE, 8 (2013) e63761. doi: 10.1371/journal.pone.0063761 23717479 PMC3661668

[11] Pacheco J.M., Traulsen A., Ohtsuki H., Nowak M.A., Repeated games and direct reciprocity under active linking, J. Theor. Biol., 250 (2008) 723–731. 10.1016/j.jtbi.2007.10.040 PMC2460569 18076911

[12] Ohtsuki H., Hauert C., Lieberman E., Nowak M.A., A simple rule for the evolution of cooperation on graphs and social networks, Nature, 441 (2006) 502–505. doi: 10.1038/nature04605 16724065 PMC2430087

[13] Schmid L., Chatterjee K., Hilbe C., Nowak M.A., A unified framework of direct and indirect reciprocity, Nat. Hum. Behav., (2021) 1–11. 33986519 10.1038/s41562-021-01114-8

[14] Whitaker R.M., Colombo G.B., Rand D.G., Indirect reciprocity and the evolution of prejudicial groups, Sci. Rep., 8 (2018) 1–14. 30185868 10.1038/s41598-018-31363-z PMC6125379

[15] Gracia-Lázaro C., Cuesta J.A., Sánchez A., Moreno Y., Human behavior in Prisoner’s Dilemma experiments suppresses network reciprocity, Sci. Rep., 2 (2012) 1–4. doi: 10.1038/srep00325 22439103 PMC3309394

[16] Wang Z., Szolnoki A., Perc M., Interdependent network reciprocity in evolutionary games, Sci. Rep., 3 (2013) 1–7. doi: 10.1038/srep01183 23378915 PMC3560361

[17] Dercole F., Della Rossa F., Piccardi C., Direct reciprocity and model-predictive rationality explain network reciprocity over social ties, Sci. Rep., 9 (2019) 1–13. 30931975 10.1038/s41598-019-41547-w PMC6443768

[18] Santos F.C., Pacheco J.M., Scale-free networks provide a unifying framework for the emergence of cooperation, Phys. Rev. Lett., 95 (2005) 098104. doi: 10.1103/PhysRevLett.95.098104 16197256

[19] Taylor P.D., Day T., Wild G., Evolution of cooperation in a finite homogeneous graph, Nature, 447 (2007) 469–472. doi: 10.1038/nature05784 17522682

[20] Allen B., Lippner G., Chen Y.-T., Fotouhi B., Momeni N., Yau S.-T., et al., Evolutionary dynamics on any population structure, Nature, 544 (2017) 227–230. doi: 10.1038/nature21723 28355181

[21] Su Q., Li A., Wang L., Eugene Stanley H., Spatial reciprocity in the evolution of cooperation, Proceedings of the Royal Society B, 286 (2019) 20190041. doi: 10.1098/rspb.2019.0041 30940065 PMC6501675

[22] Hauert C., Doebeli M., Spatial structure often inhibits the evolution of cooperation in the snowdrift game, Nature, 428 (2004) 643–646. doi: 10.1038/nature02360 15074318

[23] Lieberman E., Hauert C., Nowak M.A., Evolutionary dynamics on graphs, Nature, 433 (2005) 312–316. doi: 10.1038/nature03204 15662424

[24] Nowak M.A., Sigmund K., Evolutionary dynamics of biological games, Science, 303 (2004) 793–799. doi: 10.1126/science.1093411 14764867

[25] Du J., Wu B., Wang L., Aspiration dynamics in structured population acts as if in a well-mixed one, Sci. Rep., 5 (2015) 1–7. 10.1038/srep08014 PMC4306144 25619664

[26] Chen X, Wang L. Promotion of cooperation induced by appropriate payoff aspirations in a small-world networked game. Physical Review E, 2008, 77(1): 017103. 10.1103/PhysRevE.77.017103 18351965

[27] Liu R R, Jia C X, Rong Z. Effects of enhancement level on evolutionary public goods game with payoff aspirations. Applied Mathematics and Computation, 2019, 350: 242–248.

[28] Liu L, Wang S, Chen X, et al. Evolutionary dynamics in the public goods games with switching between punishment and exclusion. Chaos: An Interdisciplinary Journal of Nonlinear Science, 2018, 28(10): 103105. doi: 10.1063/1.5051422 30384651

[29] Liu L, Chen X. Evolutionary game dynamics in multiagent systems with prosocial and antisocial exclusion strategies. Knowledge-Based Systems, 2020, 188: 104835.

[30] Han T A. Institutional incentives for the evolution of committed cooperation: ensuring participation is as important as enhancing compliance. Journal of The Royal Society Interface, 2022, 19(188): 20220036. doi: 10.1098/rsif.2022.0036 35317650 PMC8941393

[31] Han T A, Lenaerts T. A synergy of costly punishment and commitment in cooperation dilemmas[J]. Adaptive Behavior, 2016, 24(4): 237–248.

[32] Liu Y, Chen X, Wang L, et al. Aspiration-based learning promotes cooperation in spatial prisoner’s dilemma games. Europhysics Letters, 2011, 94(6): 60002.

[33] Santos F.C., Santos M.D., Pacheco J.M., Social diversity promotes the emergence of cooperation in public goods games, Nature, 454 (2008) 213–216. doi: 10.1038/nature06940 18615084

[34] Santos F.C., Pacheco J.M., Lenaerts T., Evolutionary dynamics of social dilemmas in structured heterogeneous populations, Proceedings of the National Academy of Sciences, 103 (2006) 3490–3494. doi: 10.1073/pnas.0508201103 16484371 PMC1413882

[35] Tarnita C.E., Wage N., Nowak M.A., Multiple strategies in structured populations, Proceedings of the National Academy of Sciences, 108 (2011) 2334–2337. doi: 10.1073/pnas.1016008108 21257906 PMC3038739

[36] Su Q., Li A., Wang L., Evolutionary dynamics under interactive diversity, New J. Phys., 19 (2017) 103023.

[37] Santos F.C., Pinheiro F.L., Lenaerts T., Pacheco J.M., The role of diversity in the evolution of cooperation, J. Theor. Biol., 299 (2012) 88–96. doi: 10.1016/j.jtbi.2011.09.003 21930134

[38] Su Q., Li A., Zhou L., Wang L., Interactive diversity promotes the evolution of cooperation in structured populations, New J. Phys., 18 (2016) 103007.

[39] Su Q., Zhou L., Wang L., Evolutionary multiplayer games on graphs with edge diversity, PLoS Comput. Biol., 15 (2019) e1006947. doi: 10.1371/journal.pcbi.1006947 30933968 PMC6459562

[40] Dercole F., Della Rossa F., Piccardi C., Direct reciprocity and model-predictive rationality explain network reciprocity over social ties, Sci. Rep., 9 (2019) 5367. doi: 10.1038/s41598-019-41547-w 30931975 PMC6443768

[41] Su Q., McAvoy A., Wang L., Nowak M.A., Evolutionary dynamics with game transitions, Proceedings of the National Academy of Sciences, 116 (2019) 25398–25404. doi: 10.1073/pnas.1908936116 31772008 PMC6926053

[42] Su Q., Li A., Wang L., Evolution of cooperation with interactive identity and diversity, J. Theor. Biol., 442 (2018) 149–157. doi: 10.1016/j.jtbi.2018.01.021 29407364

[43] Jia D., Wang X., Song Z., Romić I., Li X., Jusup M., et al., Evolutionary dynamics drives role specialization in a community of players, Journal of the Royal Society Interface, 17 (2020) 20200174. doi: 10.1098/rsif.2020.0174 32693747 PMC7423431

[44] Davidai S., Ongis M., The politics of zero-sum thinking: The relationship between political ideology and the belief that life is a zero-sum game, Sci. Adv., 5 (2019) eaay3761. doi: 10.1126/sciadv.aay3761 32064320 PMC6989335

[45] Bakker B.N., Schumacher G., Gothreau C., Arceneaux K., Conservatives and liberals have similar physiological responses to threats, Nat. Hum. Behav., 4 (2020) 613–621. doi: 10.1038/s41562-020-0823-z 32042109 PMC7306406

[46] Szabó G., Tőke C., Evolutionary prisoner’s dilemma game on a square lattice, Phys. Rev. E, 58 (1998) 69.

[47] Heller Y., Mohlin E., Observations on cooperation, The Review of Economic Studies, 85 (2018) 2253–2282.

[48] Szabó G., Fath G., Evolutionary games on graphs, Phys. Rep., 446 (2007) 97–216.

[49] Traulsen A., Pacheco J.M., Nowak M.A., Pairwise comparison and selection temperature in evolutionary game dynamics, J. Theor. Biol., 246 (2007) 522–529. doi: 10.1016/j.jtbi.2007.01.002 17292423 PMC2001316

