# Supplementary figures and images for: Aspiration-driven co-evolution of cooperation with individual behavioral diversity
Source: PLoS One. 2023 Sep 15;18(9):e0291134. doi: 10.1371/journal.pone.0291134 (PMC10503719; doi:10.1371/journal.pone.0291134)

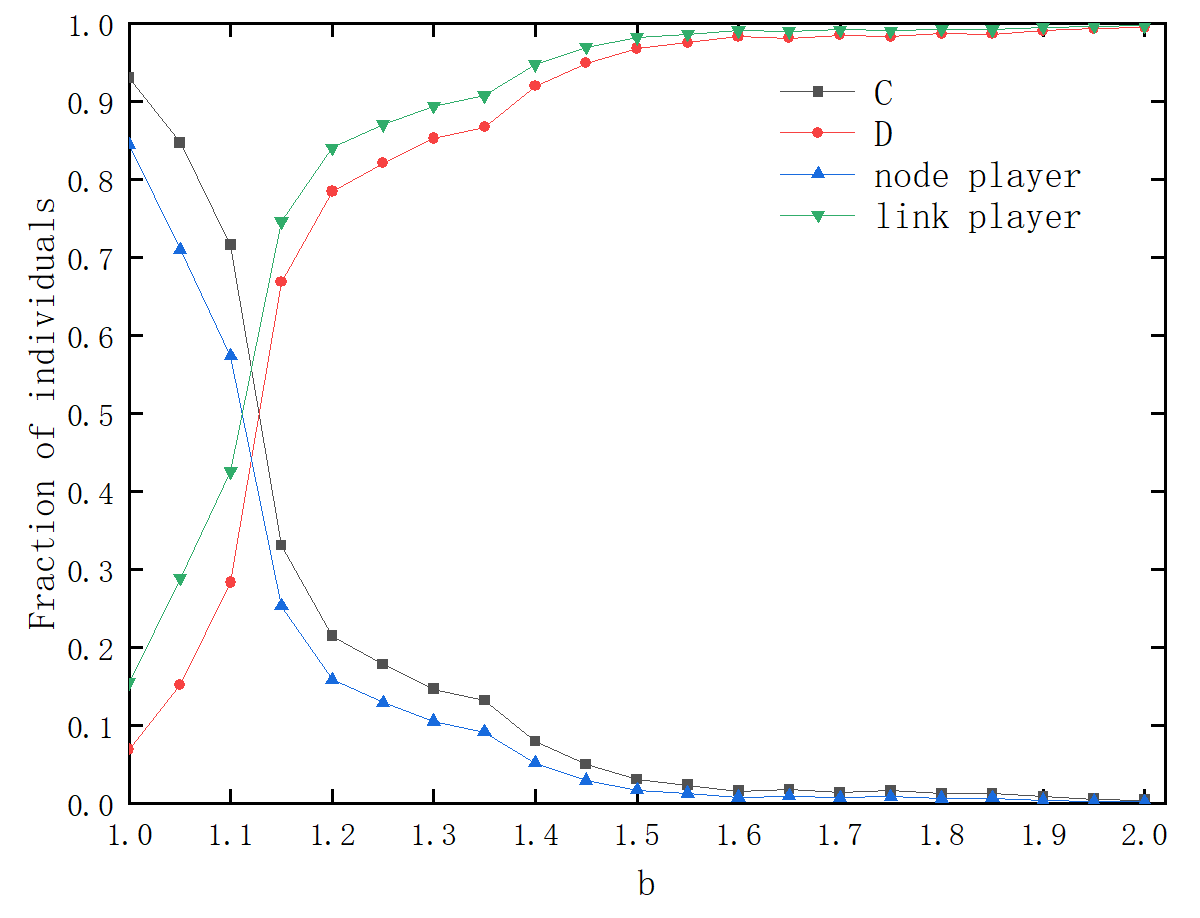

Supplement: S1 Data — (ZIP) [file pone.0291134.s001.zip › M0/v1/CD2.bmp]

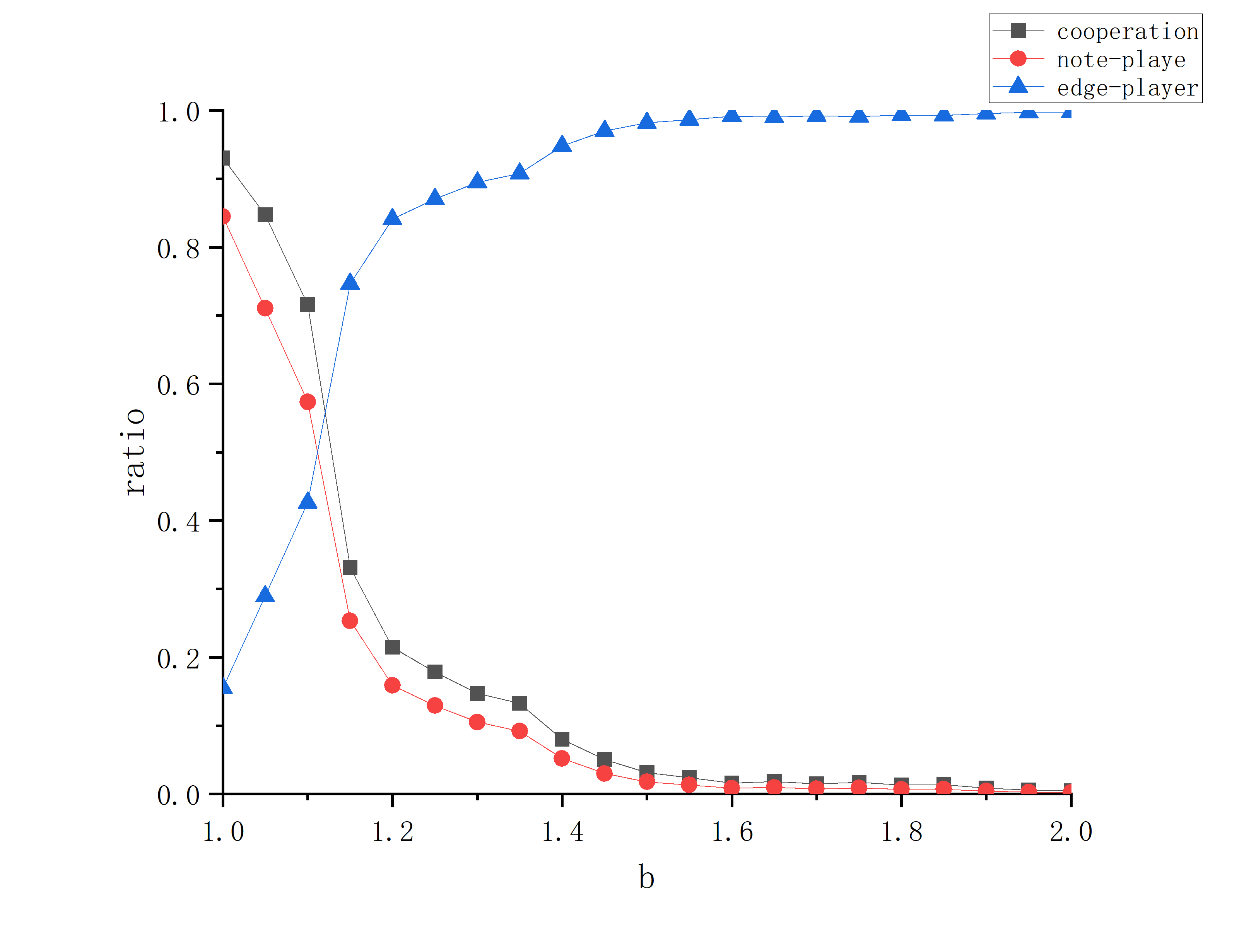

Supplement: S1 Data — (ZIP) [file pone.0291134.s001.zip › M0/v1/总览-a=0.1.bmp]

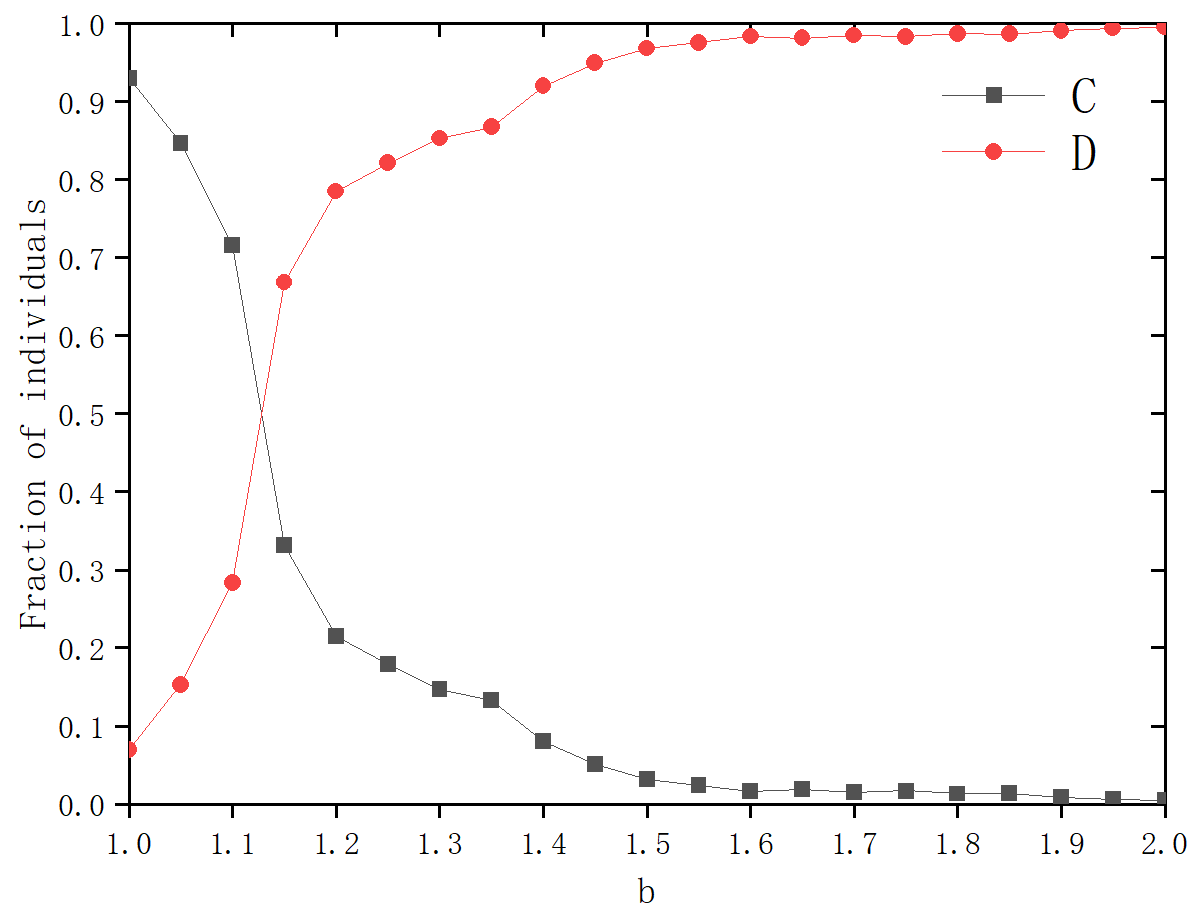

Supplement: S1 Data — (ZIP) [file pone.0291134.s001.zip › M0/v1/总览CD.bmp]

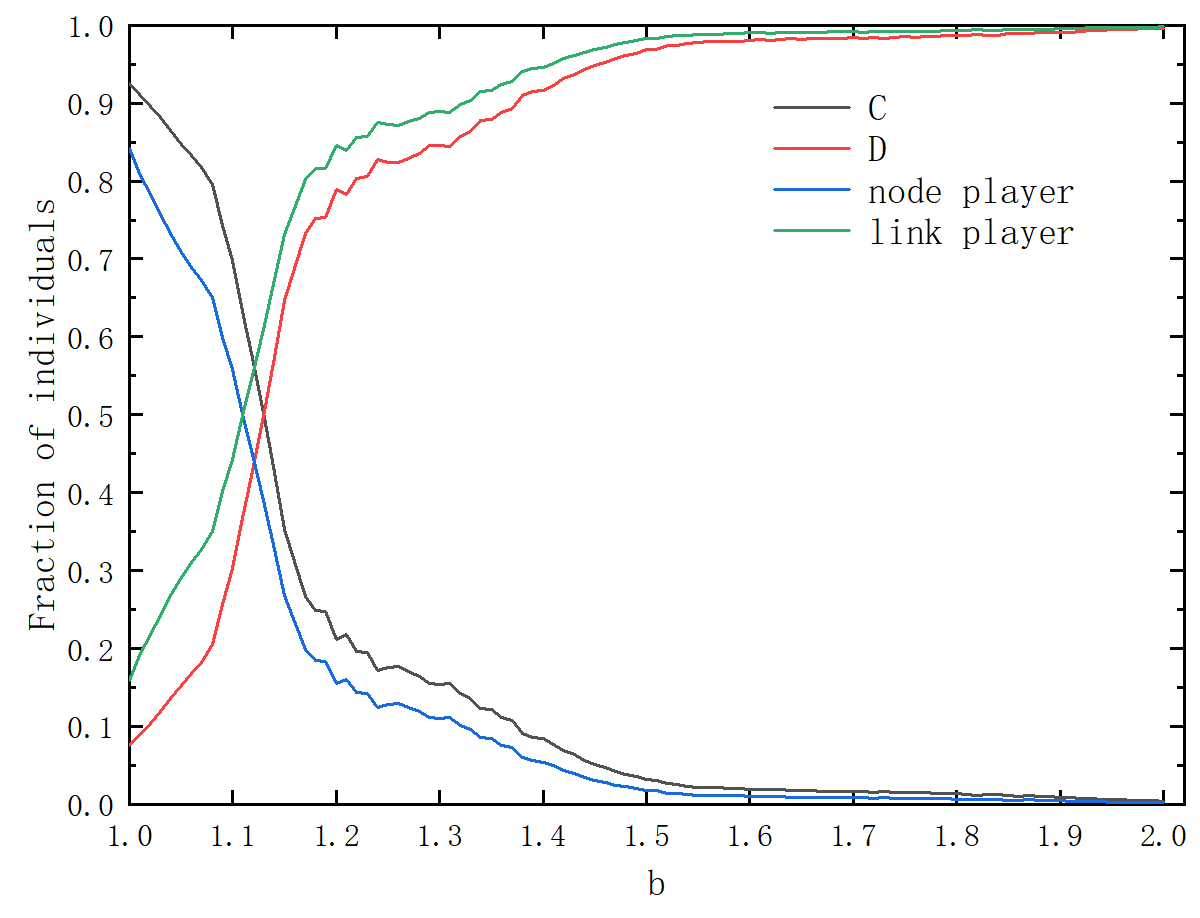

Supplement: S1 Data — (ZIP) [file pone.0291134.s001.zip › M0/v3-L=500/total.bmp]

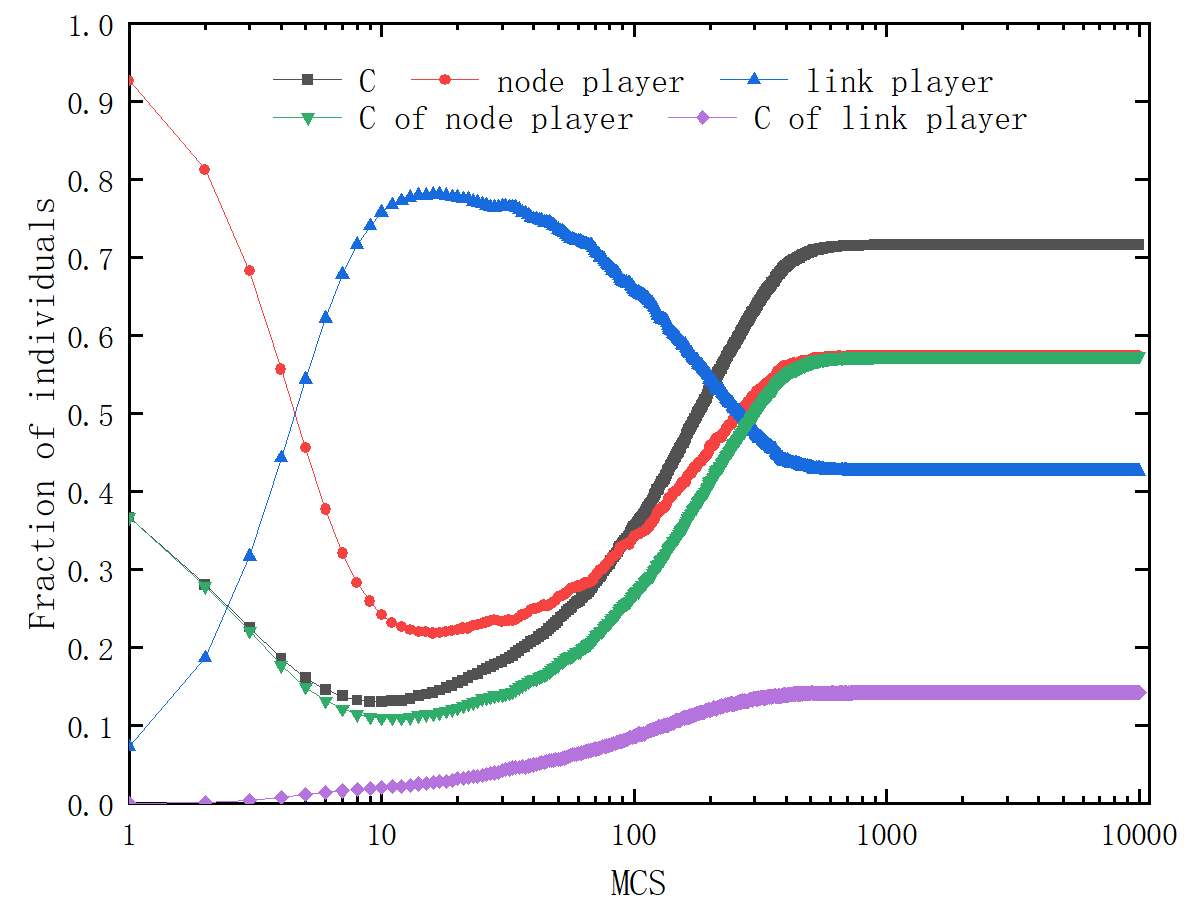

Supplement: S1 Data — (ZIP) [file pone.0291134.s001.zip › M1/v1-L=200/5线.bmp]

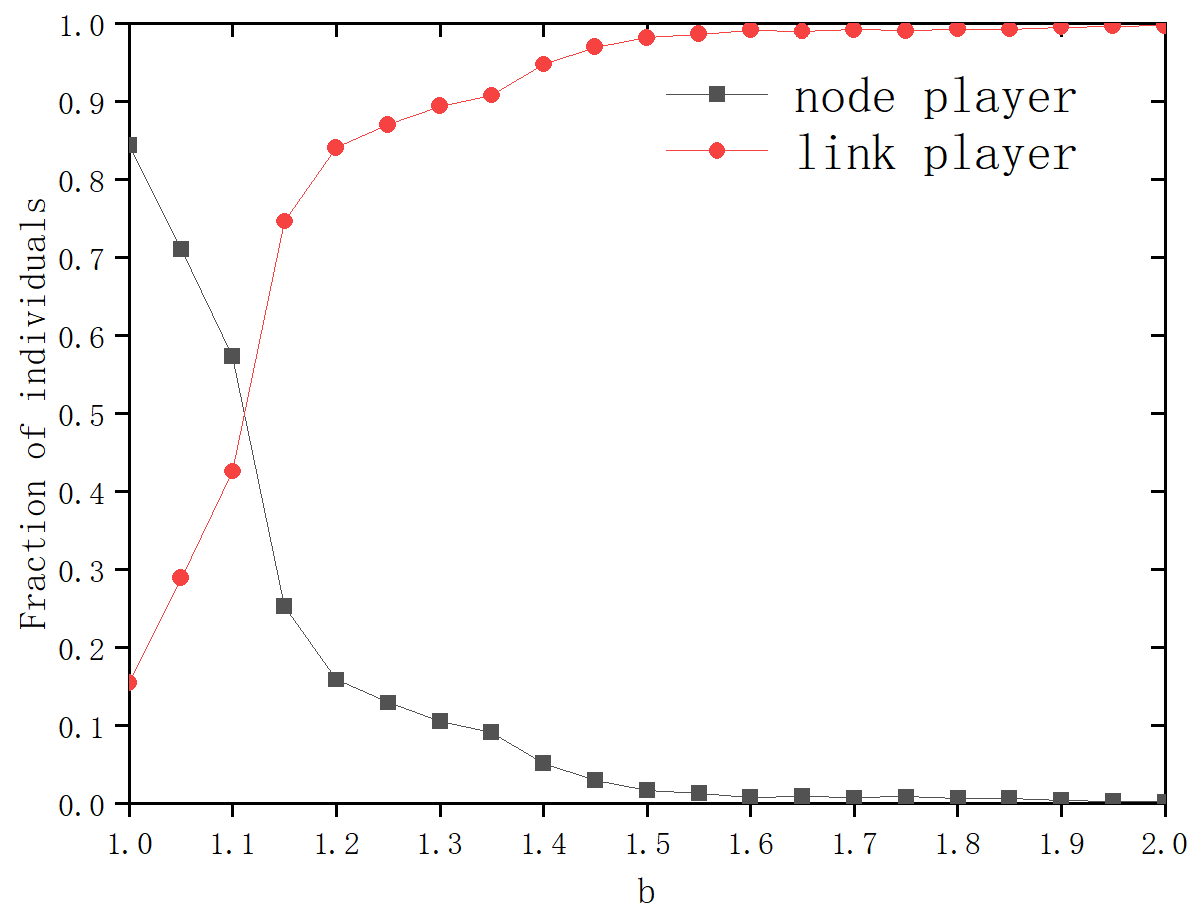

Supplement: S1 Data — (ZIP) [file pone.0291134.s001.zip › M1/v1-L=200/点边比例.bmp]

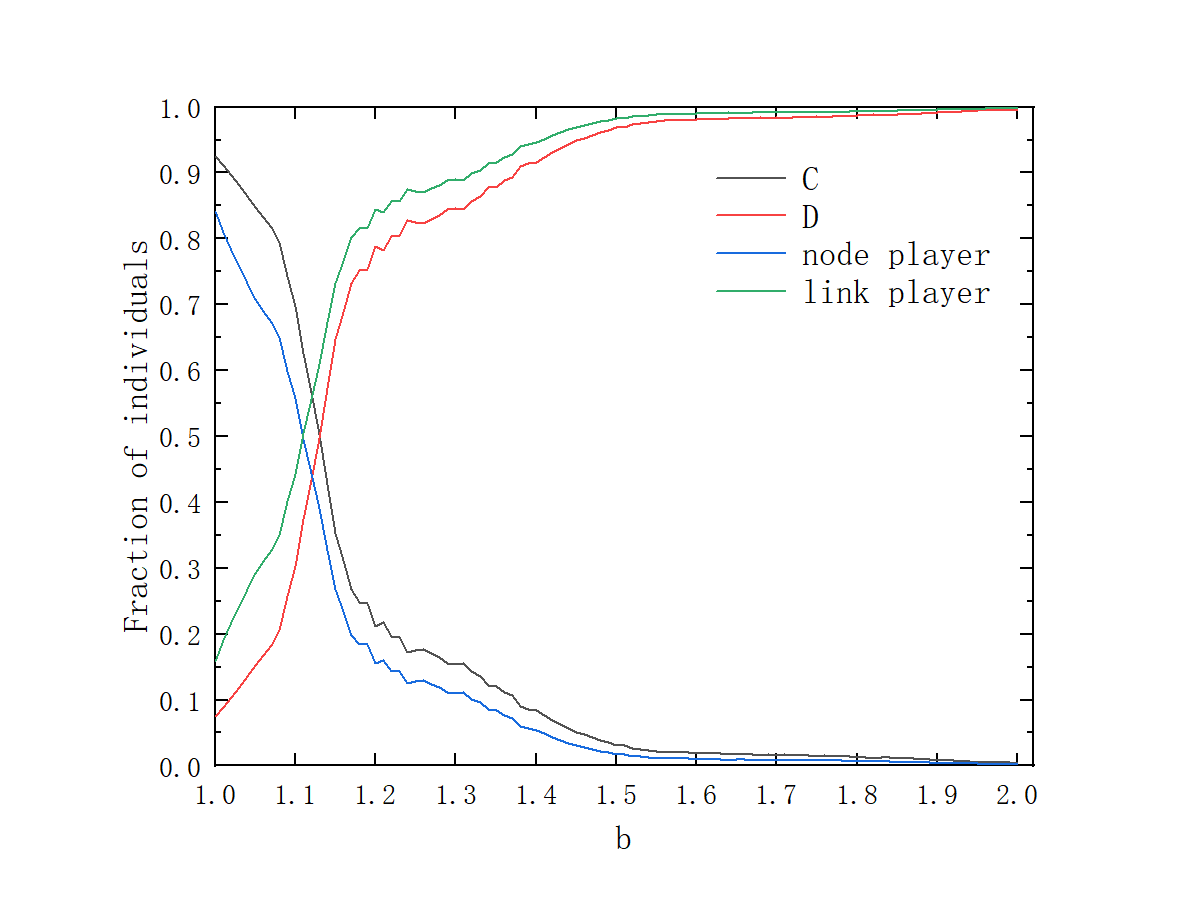

Supplement: S1 Data — (ZIP) [file pone.0291134.s001.zip › M1/v2-L=200/5线.bmp]
